# Walnut oil prevents hyperlipidemia induced by high-fat diet and regulates intestinal flora and liver metabolism

**DOI:** 10.3389/fphar.2024.1431649

**Published:** 2025-01-16

**Authors:** Rui Yang, Dan Chen, Yanling Chen, Yage Ma, Chaoyin Chen, Shenglan Zhao

**Affiliations:** ^1^ Drug and food resources development laboratory, Yunnan University of Traditional Chinese Medicine, Kunming, China; ^2^ Chemistry laboratory, Yunnan Institute of Tobacco Quality Inspection and Supervision, Kunming, China; ^3^ Modern food and tourism College cooking laboratory, Kunming University, Kunming, China

**Keywords:** walnut oil, abnormal lipid metabolism, metabolome, intestinal flora, high-fat diet

## Abstract

**Objective:**

This study aims to investigate the preventive effect of walnut oil as medicinal food on abnormal lipid metabolism and its influence on liver metabolites and intestinal flora.

**Methods:**

The rat model of abnormal lipid metabolism was established by feeding high-fat diet and administering a high-fat emulsion via gavage. The rats were randomly assigned to one of the five groups: the normal group (ND), the model group (HFD), and three walnut oil intervention groups differing in dosage [low-dose (OL, 2.5 g/kg. BW/day), medium-dose (OM, 5 g/kg. BW/day) and high-dose (OH, 10 g/kg. BW/day)]. Modeling and administration were performed simultaneously for 10 weeks. After the last administration, the serum and organs of the animals were collected under anesthesia, and the organ index was analyzed. Serum total cholesterol (TC), triglyceride (TG), low density lipoprotein cholesterol (LDL-C), high density lipoprotein cholesterol (HDL-C) were measured. A histopathological examination of the liver was performed, and the intestinal flora was detected by Illumina technology.

**Results:**

Compared to the ND group, the HFD group exhibited a significant increase in body weight and Lee’s index. Compared to the HFD group, each walnut oil intervention group showed a relatively reduced degree of liver swelling and a significant decrease in fat vacuoles within the cytoplasm. Levels of TC, TG, LDL-C, activities of alanine aminotransferase (ALT) and aspartate aminotransferase (AST) were significantly decreased (*p* < 0.05), while HDL-C levels were increased (*p* < 0.05), along with a significant increase in the activity of glutathione peroxidase (GSH-PX) and a decrease in malondialdehyde (MDA) content in serum. These findings indicated that walnut oil could improve the blood lipid profile in hyperlipidemia-model rats. The results of intestinal flora showed that at the genus level, there were significant increases in the relative abundance of *Collinsella* and *Blautia* (*p* < 0.01) while significant decreases of *Oscillospira* and *Allobaculum* (*p* < 0.01) in the HFD group vs. the ND group. However, these flora changes were impeded although only *Collinslla* (*p* < 0.05) in the OL group. Metabonomics analysis identified that a total of 19 potential biomarkers were screened out from the differential metabolites with |log_2_FC| > 1, VIP > 1 and *p* < 0.05.

**Conclusion:**

Walnut oil can significantly prevent hyperlipidemia caused by high-fat diet. The mechanism is mainly through significantly reducing the content of MDA and the activities of ALT and AST, significantly increasing the activity of GSH-PX, and improving intestinal flora and liver metabolism.

## 1 Introduction

Abnormal or disordered lipid metabolism, also known as hyperlipidemia, is a metabolic disease caused by disorder of lipid metabolism and abnormal fat transport in the body. It is characterized by high levels of total triglyceride (TG), total cholesterol (TC), low-density lipoprotein cholesterol (LDL-C) and low levels of high-density lipoprotein cholesterol (HDL-C) in serum (Chen et al., 2022). Hyperlipidemia is a significant risk factor for stroke, sudden cardiac death, hypertension, diabetes, fatty liver and obesity (Gong et al., 2023). Without regulating and treatment, it will induce atherosclerosis, liver cirrhosis, coronary heart disease, myocardial infarction, as well as other cardiovascular and cerebrovascular diseases, posing a serious threat to human health (Zhao et al., 2022). Data from the World Health Organization indicate that fatalities from hyperlipemia-related diseases account for approximately 30% of all global deaths (Yan et al., 2021). The incidence of hyperlipidemia continues to rise, affecting even younger individuals, with the dyslipidemia rate of Chinese adults reaching as high as 40.40% (Zhao et al., 2020). Hyperlipidemia has seriously affected the health of Chinese people and has become a major public health problem affecting the economic and social development of the country (Dou and Chen, 2022). Statins, the most prescribed lipid-lowering drugs, can cause adverse effects such as gastrointestinal reactions, liver function damage, and cardiac rhabdomyolysis, with an increased risk when co-administered with fibrates. Thus, dietary approaches to prevent abnormal lipid metabolism are of significant importance (Zhao et al., 2023).

The walnut kernel, documented in the pharmacopoeia as a traditional Chinese medicine, is rich in oil, unsaturated fatty acids (UFAs), polyphenols, phytosterols, squalene, vitamins, minerals and other beneficial nutrients. These components confer both medicinal and nutritional value (Joe and Cai, 2012), offering potential benefits such as the prevention of coronary heart disease, softening of blood vessels, cholesterol reduction, antioxidant effects, among others (Xu et al., 2023; Deng et al., 2022). The oil content of walnut meat is up to 60%–70% (Geng et al., 2021). Studies have shown that the polyphenolic compounds present in walnut oil have anti-inflammatory and free radical scavenging properties, thus potentially benefiting cardiovascular diseases, atherosclerosis and metabolic syndrome (Fregapane et al., 2020). In addition, Liu et al. found that α-linolenic acid inhibited platelet aggregation and thrombosis by reducing levels of blood lipids, cholesterol and low-density protein cholesterol, thereby preventing atherosclerosis and its complications (Liu et al., 2022).

In this study, hyperlipidemia-model rats with liver metabolomics and gut microbiota were used to investigate the effects and mechanisms of walnut oil in preventing abnormal lipid metabolism, so as to enhance the scientific understanding of walnut oil’s value and support its medicinal food use in the prevention of dyslipidemia.

## 2 Materials and methods

### 2.1 Materials and instruments

#### 2.1.1 Materials

Walnut oil: Dexin Kangbang Oil Industry Ltd.; Normal saline: Kunming Nanjiang Pharmaceutical Ltd.; Cholesterol: Beijing Solaibao Technology Ltd.; Egg yolk powder: buy eggs to make their own; Tween-80, RON, Item No. 9006-65-6; Pig bile Salt, Bioed, Product No. B0029D; Lard, Sichuan Kuohai Food Technology Ltd.; Propylthiouracil: Shanghai Macklin Biotechnology Ltd., Item No. P815828; 4% paraformaldehyde tissue fixative: Wuhan Sevier Biotechnology Ltd.

Experimental animals: Forty-five clean grade male Sprague-Dawley rats, weighing about (160 ± 10) g, were purchased from Hunan Slaike Jingda Laboratory Animal Ltd., qualification certificate No. SCXK (Xiang) 2019-0004. This experiment was approved by the Experimental Animal Ethics Committee of Yunnan University of Traditional Chinese Medicine (R-062023078).

High-fat feed: 60.3% base feed, 15% sucrose, 10% egg yolk powder, 12% lard, 1% pig bile salt, 1.5% cholesterol, 0.2% salt, mixed according to the formula and dried at 45°C. High fat emulsion: 15% Tween, 20% lard, 5% sugar, 6% cholesterol, 1% sodium cholate, 0.2% propyl thiouracil, and appropriate amount of distilled water, according to the ratio of confluxing stored at −4 °C.

TC assay kit, Batch number: A111-1-1; TG assay kit, Batch number: A110-1-1; LDL-C assay kit, Batch number: A133-1-1; HDL-C Assay Kit, Batch number: A112-1-1; ALT assay kit, Batch number: C009-2-1; AST assay kit, Batch number: C010-2-1; GSH-PX assay kit, Batch number: A005-1-2; MDA assay kit, batch number: A003-1-2.

#### 2.1.2 Instruments

H1850-R refrigerated centrifuge was purchased from Hunan Xiangyi Laboratory Instrument Development Ltd (Hunan, China); INFINITEM200PRO microplate reader was purchased from TECAN (Switzerland); JA-20001 electronic analytical balance was purchased from Guangzhou Yuzhi Instrument Ltd. (Guangzhou, China). MB-96 high-throughput tissue grinder was purchased from Zhejiang Meibi Instrument Ltd. (Zhejiang, China), QL-866 vortex mixer was purchased from Haimen Qilinbeier Instrument Manufacturing Ltd. (Jiangsu, China), KQ-800DE ultrasonic cleaner was purchased from Kunshan Ultrasonic Instrument Ltd. (Jiangsu, China), QL-866 vortex mixer was purchased from Haimen Qilinbeier Instrument Manufacturing Ltd. The 5305 vacuum concentrator was purchased from Abendt AG (Germany), 0.22 µm PTFE filter membrane was purchased from Tianjin Jinteng Technology Ltd. (Tianjin, China), TRACE 1300 gas chromatoscope and ISQ 7000 mass spectrometer were purchased from Thermo Fisher Scientific (Massachusetts, United States).

### 2.2 Experimental methods

#### 2.2.1 Grouping and treatment of experimental animals

Clean-grade male SD rats were adaptively fed in an environment of 22°C–25°C, 60% relative humidity and 12 h circadian ratio for 1 week. Then they were randomly divided into normal group (ND), model group (HFD), walnut oil low-dose prevention group (OL), walnut oil medium-dose prevention group (OM) and walnut oil high-dose prevention group (OH). There was no significant difference in body weight among the groups (*p* > 0.05), with 9 rats in each group. The normal group was fed with basic diet, the model group and the prevention group were fed with high-fat diet and high-fat emulsion by gavage, and the high-fat emulsion was given 1 mL/100 g by gavage every 3 days. The death, diet water, feces, spirit and hair of the rats were observed. Body weight was measured once a week and recorded for 10 weeks.

The dosage of walnut oil by gavage was according to the recommended dose in the “Dietary Guidelines for Chinese Residents (2022)”, and the recommended dose of walnut oil was 25 mL per person per day, which was calculated according to the conversion factor of rat. The low dose of walnut oil was 2.5 mL/kg. BW, the middle dose was 5 mL/kg. BW, and the high dose was 10 mL/kg. BW (the dosage was adjusted according to the state and body weight of the rats).

#### 2.2.2 Determination of body mass and Lee’s index of rats

After gavage, all rats were fasted for 12 h without drinking. The body weight and length of the rats were measured the next day. Blood samples were collected from the abdominal aorta after anesthesia with 10% pentobarbital.

$${\text{Lee}}^{\prime }s\text{ index}={\left\{\text{body}\;\text{weight}\;\left(g\right)\times 1000/\;\text{body}\;\text{length}\;\left(\text{cm}\right)\right\}}^{1/3}$$

#### 2.2.3 Determination of serum biochemical indexes and organ weight of rats

The blood samples were left at 4°C for 2 h and centrifuged at 3,500 r/min for 15 min. The non-hemolytic serum samples were collected to determine the levels of TC, TG, HDL-C, LDL-C, MDA, AST, ALT, and GSH-PX in the serum samples of rats by the corresponding kits. After the rats were anesthetized, the whole liver, spleen and kidney of the rats were quickly removed, and the perirenal and testicular adipose tissues were dissected, and the organ index and fat index were calculated.

$$\text{Liver index}=\text{liver mass }\left(g\right)/\text{rat body mass }\left(g\right)\times 100\;\%$$

$$\text{Spleen index}=\text{spleen mass }\left(g\right)/\text{rat body mass }\left(g\right)\times 100\;\%$$

$$\text{Kidney index}=\text{kidney mass }\left(g\right)/\text{rat body mass }\left(g\right)\times 100\;\%$$

$$\text{Perirenal fat index}=\text{perirenal fat mass }\left(g\right)/\text{rat body mass }\left(g\right)\times 100\;\%$$

#### 2.2.4 Sample sampling and pretreatment

##### 2.2.4.1 Liver tissue pretreatment

Part of the liver lobular tissue was fixed with 4% paraformaldehyde solution, rinsed with running water, dehydrated, and embedded in clear wax. The tissue was cut into 5–8 μm tissue slides, attached to the slide, stained with hematoxylin and eosin, and then fixed for histopathological observation.

##### 2.2.4.2 Liver metabolite extraction pretreatment

Accurately weigh an appropriate amount of samples into a 2 mL centrifuge tube, accurately add 1,000 µL tissue extract [75% (9:1 methanol:chloroform): 25% H_2_O], and add steel beads;

1) Put them into a tissue grinder and grind for 60 s at 50 Hz, then repeat the above operation twice.
2) Ultrasound at room temperature for 30 min and place on ice for 30 min;
3) Centrifuged at 12,000 rpm and 4°C for 10 min. The supernatant was taken into a centrifuge tube, concentrated and dried.
4) Accurately add 200 μL of 50% acetonitrile solution to configure 2-chloro-L-phenylalanine solution (4 ppm) to redissolve the sample, and add the filtrate to the detection bottle for LC-MS detection (Curtis R et al., 2017).

#### 2.2.5 Analysis of intestinal flora

The colonic contents of rats were snap-frozen in liquid nitrogen and frozen at −80°C for sequencing of intestinal flora.

Total DNA was extracted from rat intestinal contents samples, quantified by Nanodrop, and the quality of DNA extraction was detected by 1.2% agarose gel electrophoresis. The recovered PCR products were quantified by fluorescence using Quant-iT PicoGreen dsDNA Assay Kit and Microplate reader (BioTek, FLx800). Paired-end sequencing analysis of gut microbiota samples was performed on Illumina platform. Sequencing libraries were prepared by TruSeq Nano DNA LT Library Prep Kit and quantified on Promega QuantiFluor system after quality control. Qualified sequencing libraries were diluted and mixed according to the required sequencing amount. High-throughput sequencing was performed. The level of alpha diversity was assessed for each sample based on the distribution of ASV/OTU in different samples. At the species taxonomic composition level, the differences in species abundance composition between different groups were analyzed.

#### 2.2.6 Metabolomics detection

##### 2.2.6.1 Chromatographic conditions

Thermo Vanquish (Thermo Fisher Scientific, United States) Ultra Performance Liquid System, An ACQUITY UPLC^®^ HSS T3 (2.1 × 100 mm, 1.8 µm) (Waters, Milford, MA, United States) column was used at a flow rate of 0.3 mL/min, column temperature of 40°C, and injection volume of 2 μL. In positive ion mode, the mobile phase was 0.1% formic acid acetonitrile (B2) and 0.1% formic acid water (A2), and the gradient elution program was as follows: 0–1 min, 8% B2; 1–8 min, 8%–98% B2; 8–10 min, 98% B2; 10–10.1 min, 98%–8% B2; 10.1–12 min, 8% B2. In negative ion mode, the mobile phase was acetonitrile (B3) and 5 mM ammonium formate water (A3), and the gradient elution program was as follows: 0–1 min, 8% B3; 1–8 min, 8%–98% B3; 8–10 min, 98% B3; 10–10.1 min, 98%–8% B3; 10.1–12 min, 8% B3 (Eva Z et al., 2009).

##### 2.2.6.2 Mass spectrum conditions

Thermo Orbitrap Exploris 120 mass spectrometer detector (Thermo Fisher Scientific, United States), electrospray ion source (ESI), and data were collected separately in positive and negative ion modes. The positive ion spray voltage was 3.50 kV, the negative ion spray voltage was −2.50 kV, the sheath gas was 40 arb, and the auxiliary gas was 10 arb. The capillary temperature was 325°C, the first stage full scanning was performed with a resolution of 60,000, the first stage ion scanning range 100–1,000, and the second stage lysis was carried out with HCD, the collision energy 30%, the second stage resolution 15,000, and the four ions were cleaved before the signal was collected. At the same time, dynamic exclusion was used to remove unnecessary MS/MS information (Elizabeth J et al., 2013).

##### 2.2.6.3 Differential metabolite analysis

The metabolites were identified by high-resolution MS/MS spectral data which were matched with HMDB (http://www.hmdb.ca), massbank (http://www.massbank.jp/), LipidMaps (http://www.lipidmaps.org), mzcloud (https://www.mzcloud.org) and the metabolite database established by Panomix Biomedical Tech Co., Ltd (Shuzhou, China). Using the Majorbio Cloud Platform for data analysis, the differential metabolites were analyzed by orthogonal partial least squares-discriminant analysis (OPLS-DA). The significantly different metabolites were selected based on the OPLS-DA model’s variable importance in projection (vip>1) score, *p-*value < 0.05 of univariate statistical analysis and fold change >2 (i.e., |log_2_FC| > 1). Using the KEGG pathway database (https://www.kegg.jp/pathway.html), the pathways associated with the differential metabolites were obtained.

#### 2.2.7 Data processing

Test data were expressed as mean ± standard deviation, processed by GraphPadPrism 8.0.2 statistical software, processed by one-way variance, and compared between two groups by Dunnett-t test, *p <* 0.05 was considered significant.

## 3 Results and analysis

### 3.1 Measurement of body weight and Lee’s index of rats

**FIGURE 1:**
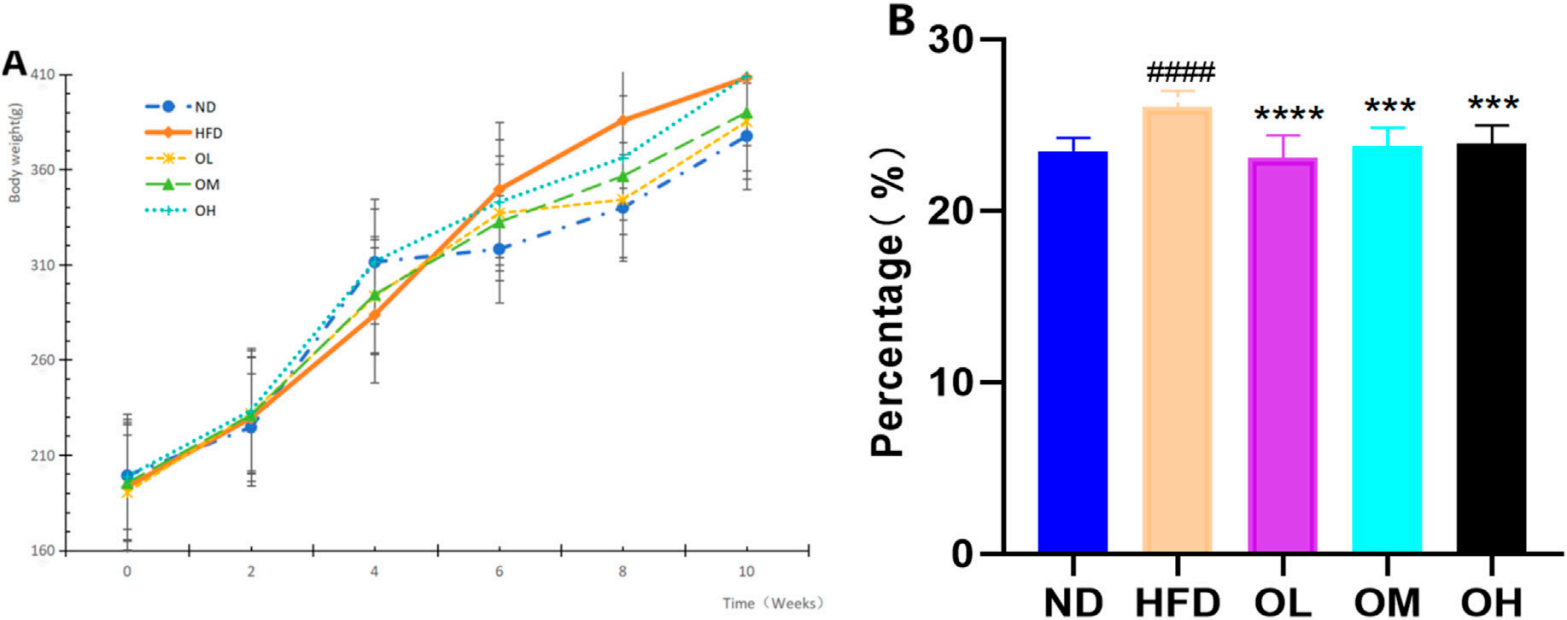
Effects of walnut oil on body weight and Lee’s Index in rats. **(A)** Body weight versus time analysis; **(B)** Lee’s index. Note: compared with group ND, ####: *p* < 0.0001; Compared with group HFD, ***: *p* < 0.001, ****: *p* < 0.0001.

During the modeling and administration period, the changes in body weight of rats in each group were recorded, depicted in Figuer 1A. The rats in ND group were active with no mortalities and maintained a regular diet and exhibited a continued increase in body weight. Similarly, the rats in HFD group experienced no fatalities, but their body weight escalated rapidly. There was no death in the other treatment groups, with normal diet and no abnormalities in bowel and urine. Before the administration, there was no significant difference in the body weight of rats in each experimental group (*p* > 0.05). After administration, the body weight of rats in each experimental group showed varying upward trend. By the sixth week following the termination of the experiment, the weight gain rates were as follows: 86.54% for the ND group, 114.66% fpr the HFD group, 88.94% for the OL group, 100% for the OM group, and 77.83% for the OH group. Compared to the ND group, the Lee’s index of the HFD group registered a significant increased (*p* < 0.0001), indicative of fat accumulation due to the high-fat diet. Conversely, when compared to the HFD group, the Lee’s index of the rats in each intervention group showed a marked decreased (*p <* 0.001). Walnut oil effectively prevented weight gain in rats subjected to a high-fat diet and exerted a pronounced preventive effect on hyperlipidemia, as illustrated in Figure 1B.

### 3.2 Effects on organ indices of rats

**FIGURE 2:**
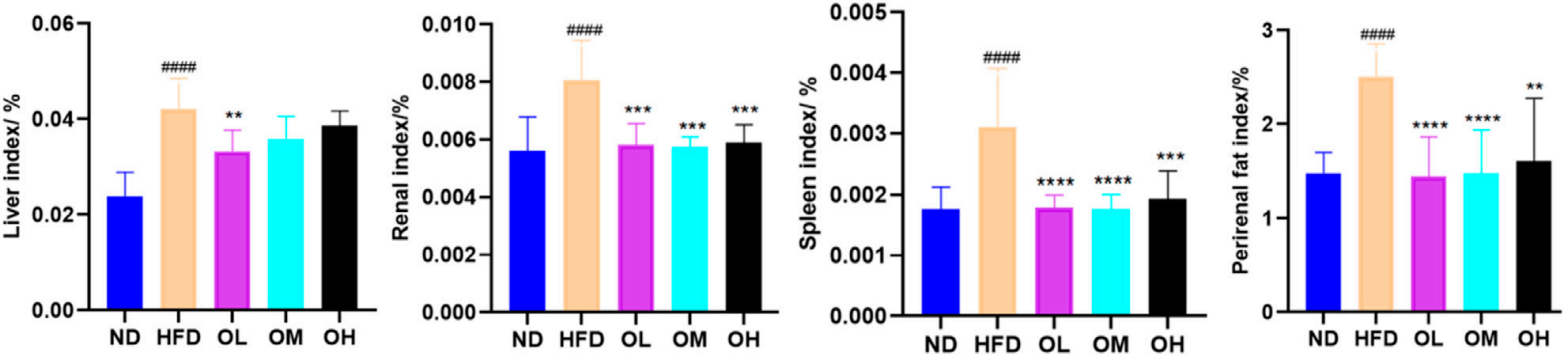
Effect of walnut oil on organ body weight in rats. Note: compared with group ND, ###: *p* < 0.001, #####: *p* < 0.0001; Compared with group HFD, **: *p* < 0.01, ***: *p* < 0.001,****: *p* < 0.0001.

A high-fat diet significantly increased the indices of liver, spleen, kidney and perirenal fat (*p* < 0.01). Simultaneous gavage of walnut oil notably mitigated the increases in spleen, kidney and perirenal fat indices (*p* < 0.01), although reduced the liver index only in the OL group (*p* < 0.01), as shown in Figure 2.

### 3.3 Effects on serum biochemical parameters of rats

**FIGURE 3:**
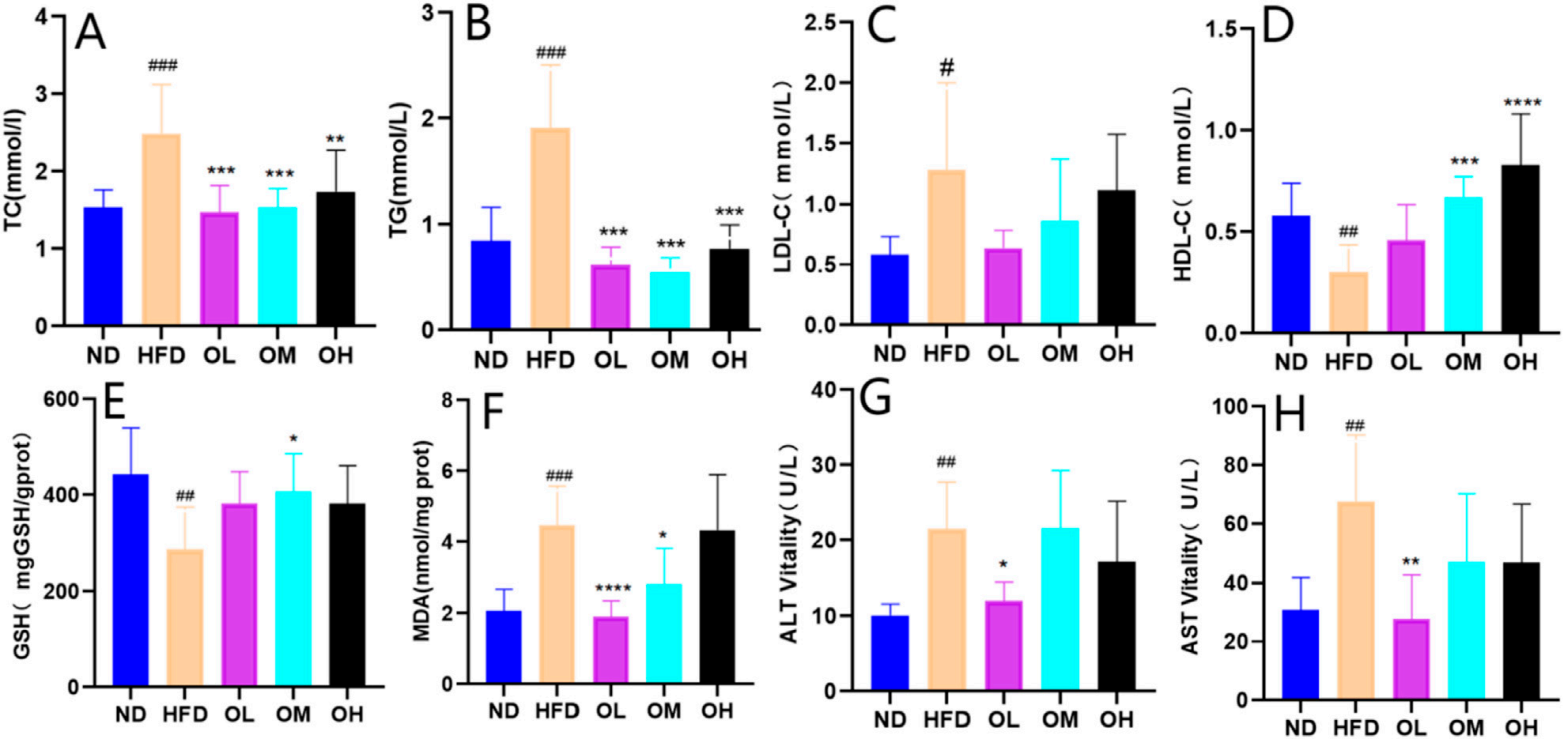
Effect of walnut oil on serum biochemical indexes of rats. **(A–H)** Serum levels of TC, TG, LDL-C, HDL-C, GSH-PX, MDA, ALT and AST. Note: compared with group ND, #: *p* < 0.05, ##: *p* < 0.01, ###: *p* < 0.001; Compared with group HFD, *: *p* < 0.05, **: *p* < 0.01, ***: *p* < 0.001, ****: *p* < 0.0001.

The effect of walnut oil on blood lipid was illustrated in Figures 3A–D. Compared to the ND group, the HFD group exhibited increased serum levels of TG, TC and LDL-C, while HDL-C levels were significantly decreased (*p* < 0.05). Compared with HFD group, the serum levels of TG, TC and LDL-C levels were lower, and HDL-C levels were higher significantly (*p* < 0.01) in walnut oil group.

The effects of walnut oil on serum levels of GSH-PX and MDA were shown in Figures 3E, F. Compared to the ND group, serum GSH-PX in HFD group was significantly decreased (*p* < 0.01) and MDA was significantly increased *(p <* 0.001). Compared to the HFD group, the GSH-PX levels in walnut oil groups were higher, with significant differences in OM group (*p* < 0.05). Similarly, the MDA levels in the OL and OM group were significantly lower (*p < 0.05*). These results indicated that walnut oil enhanced serum glutathione peroxidase and decreased the of malondialdehyde levels, conferring antioxidant benefits.

The effects of walnut oil on serum liver function indices ALT and AST levels were shown in Figures 3G, H. Compared to ND group, the serum ALT and AST levels of rats in HFD group were significantly increased (*p* < 0.05). However, compared with the HFD group, these levels in walnut oil groups were lower, with the OL group showing a significant difference (*p* < 0.05). These results indicated that walnut oil prevented from the increase of serum levels of ALT and AST in hyperlipidemia rats, thereby exerting a protective effect on the liver, a crucial organ in lipid metabolism (Yan et al., 2023).

### 3.4 Results of pathological sections of rat liver

**FIGURE 4:**
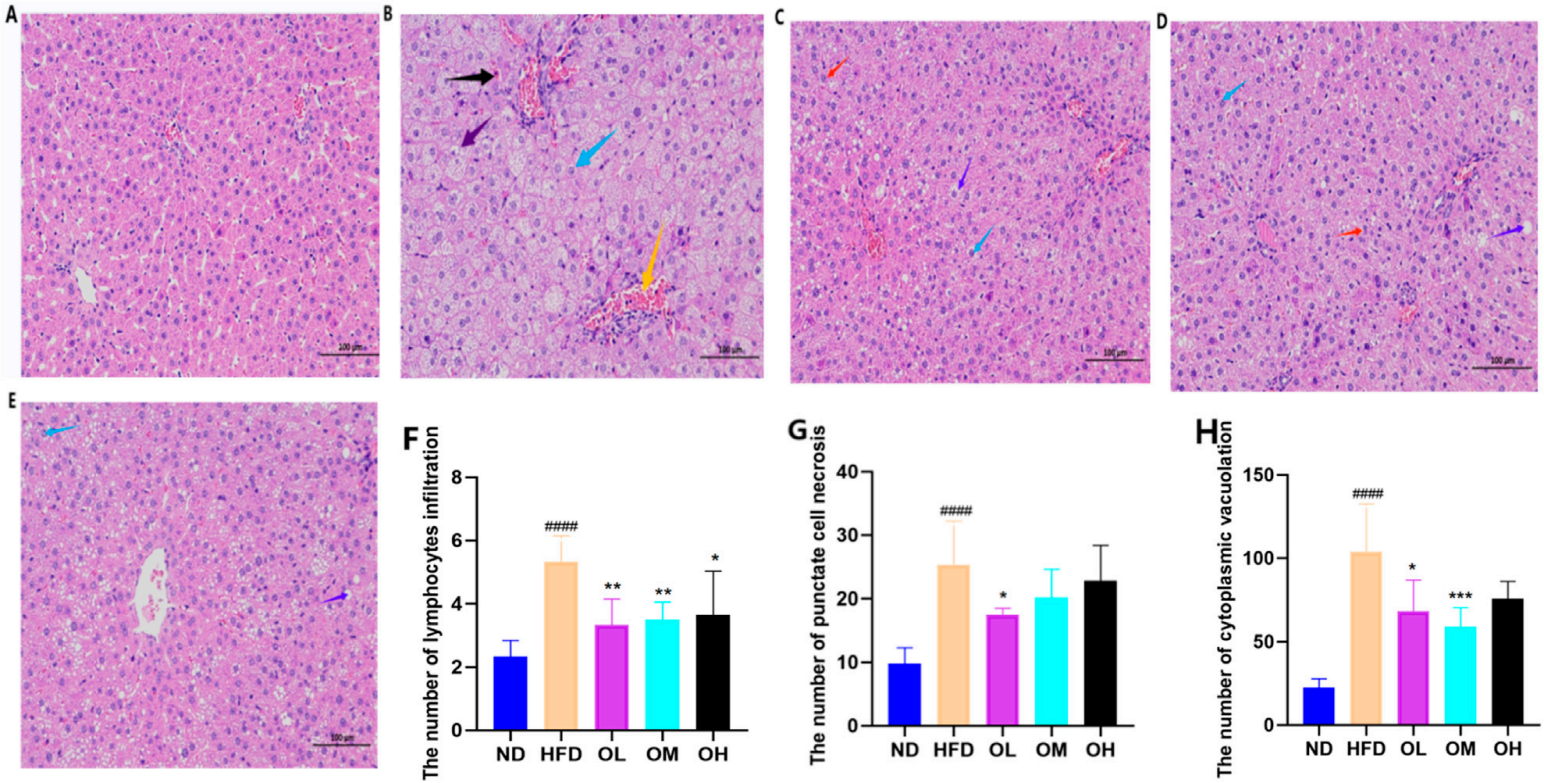
Effect of walnut oil on liver tissue morphology of rats with high-fat diet (×200). **(A–E)** HE staining of liver tissue sections was divided into normal group, model group, walnut oil low-dose group, walnut oil medium dose group, and walnut oil high-dose group. **(F–H)** Quantitative analysis of liver punctate necrosis, lymphocyte infiltration and cytoplasmic vacuolation. Note: compared with group ND, ###: *p* < 0.001; Compared with group HFD, *: *p* < 0.05, **: *p* < 0.01, ***: *p* < 0.001.

Consumption of high-fat diet can cause liver steatosis and inflammation. As shown in Figures 4A–E, the liver tissue structure in the ND group was intact, with regular liver lobules and neatly arranged hepatocytes. In the center of the hepatic lobules, the central vein was prominently visible, surrounded by hepatocytes and hepatic blood sinuses in a radial configuration. In the HFD group, the irregular arrangement and the extensive steatosis (marked by blue arrows) of hepatocytes were observed. The small round vacuoles were observed in the cytoplasm, along with a large amount of balloon-like transformation (marked by purple arrows) and enlargement. A small amount of lymphocyte infiltration (marked by orange arrows) were found around a few of the portal areas, with occasional spot-like necrosis of hepatocytes (marked by black arrows), as well as the nucleolar shrinkage and fragmentation, indicating the pro-steatotic and pro-inflammatory effects of a high-fat diet. Compared with HFD group, the OL, OM and OH groups displayed hepatocyte disarray, cell swelling, and loose cytoplasm with light staining. A small amount of severe hepatocyte changes of ballooning (purple arrow), enlargement, and steatosis (blue arrow) were noted, with small circular cytoplasmic vacuoles and extensive edema of hepatocytes (red arrow) in OL and OM groups (He et al., 2024). Liver spot-like necrosis, lymphocyte infiltration, and cytoplasmic vacuolation were significantly different in the OL group (*p* < 0.05), as shown in Figures 4F, G.

### 3.5 Intestinal flora characteristics of rats in each group

#### 3.5.1 Alpha diversity analysis

**FIGURE 5:**
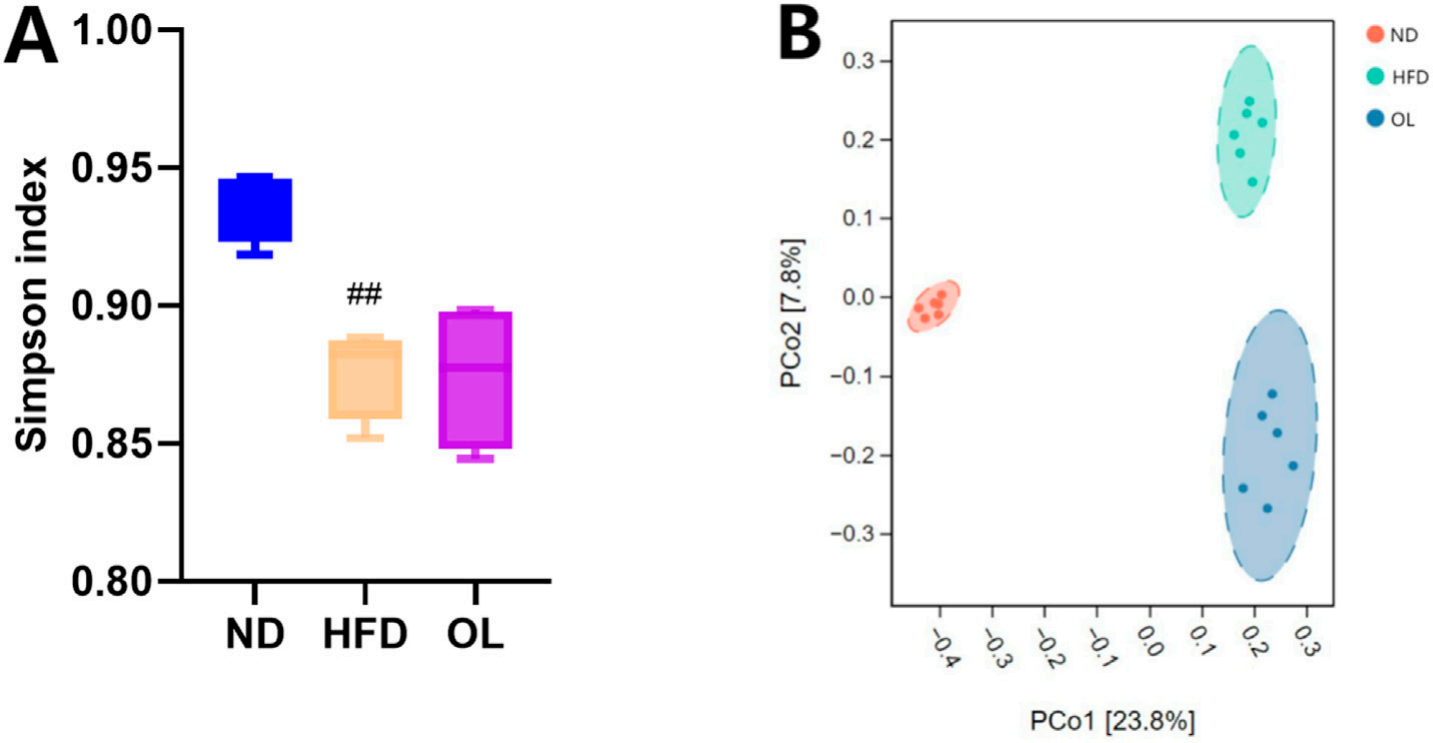
Biodiversity assessmentof gut microbiota among the ND, HFD, and OL groups (n = 6). **(A)** Simpson index; **(B)** PCoA plot. Note: compared with group ND, ##: *p* < 0.01.

Alpha diversity refers to the richness, diversity and evenness of species in locally homogeneous habitats, also known as in-range diversity. Alpha diversity, which represented in-habitat diversity, was assessed using the Simpson index in this study. Compared to the ND group, the Simpson index of HFD group was significantly decreased (*p* < 0.05). In contrast, the OL group showed a notable correction of the Simpson index relative to the HFD group, as illustrating in Figure 5A.

#### 3.5.2 Beta diversity analysis

The PCoA analysis was based on the binary_jaccard distance, and the results were shown in Figure 5B. The sample points within each group were clustered in the same confidence interval, and the sample points were significantly separated between each group, indicating that there was a significant difference in β diversity of intestinal flora among the groups. Compared with the ND group, the samples in the OL group and the HFD group were distributed closer but without overlapping regions, indicating that walnut oil intervention can still change the structure of intestinal flora in rats with high-fat diet to a certain extent.

#### 3.5.3 Amplicon sequence variant (ASV)/operational taxonomic unit (OTU) wayne diagram

**FIGURE 6:**
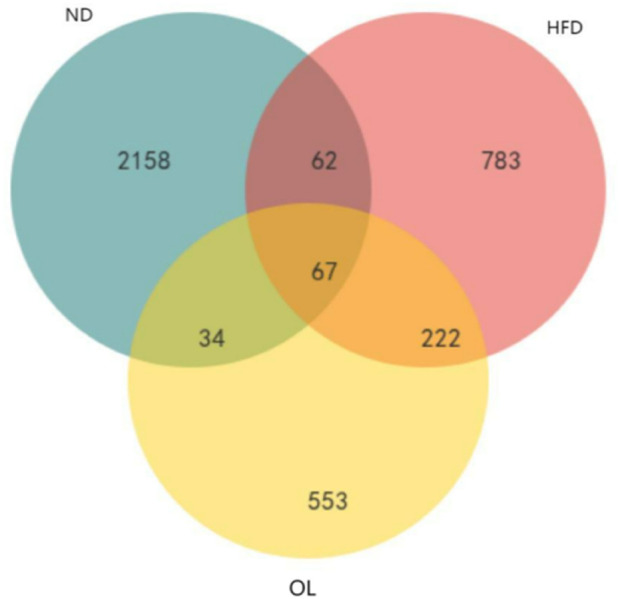
Venn diagram of common functional groups.

Venn diagram results were showed in Figure 6. A total of 67 OTUs were common across all three groups, while 129 OTUs were in common between ND and HFD groups and 289 OTUs were in common between HFD and OL. The substantial proportion of unique OTUs in each group suggested significant variations in bacterial community composition among the groups.

#### 3.5.4 Analysis of intestinal community composition

**FIGURE 7:**
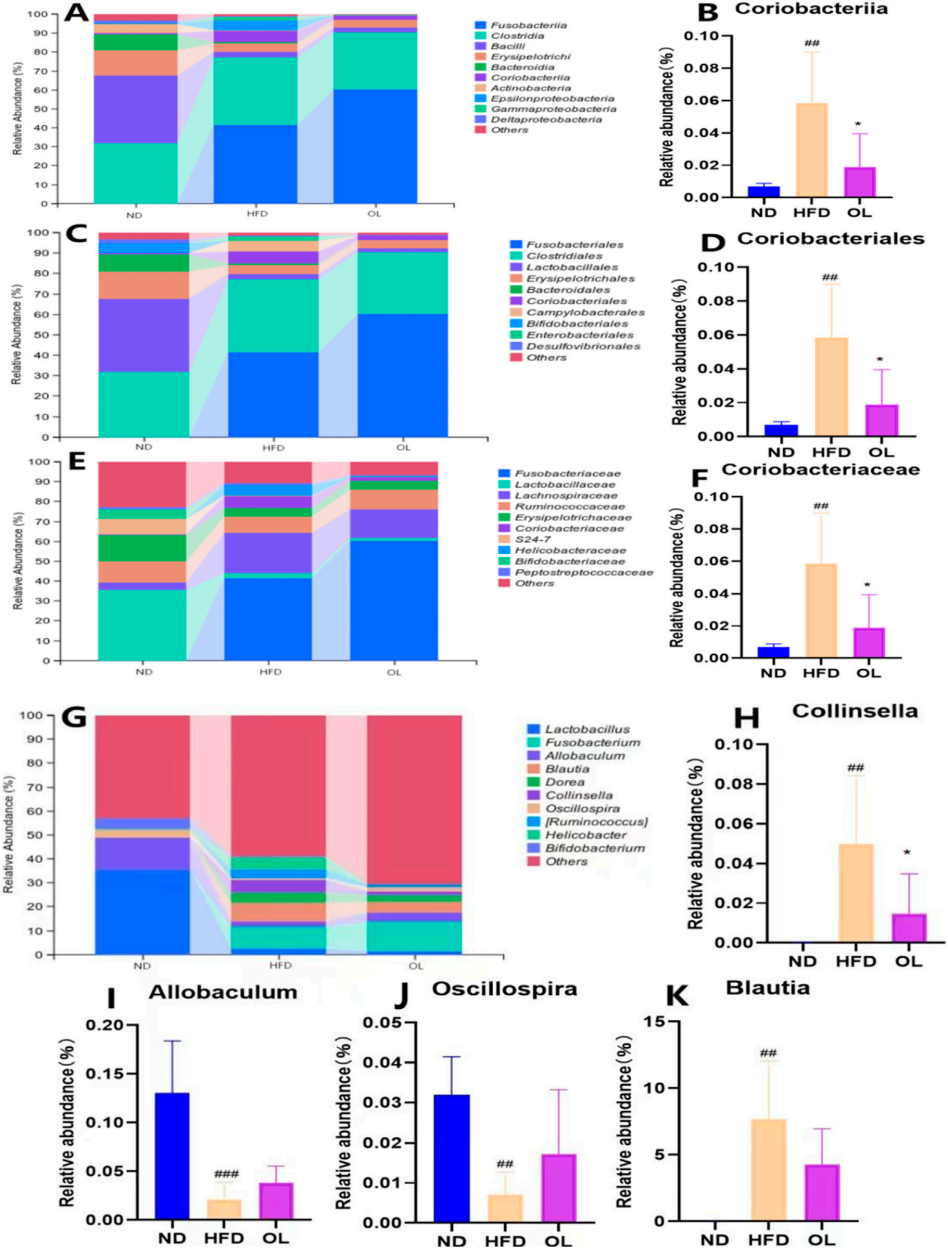
Changes in gut microbiota among the ND, HFD, and OL groups at different taxonomic levels (n = 6). **(A)** The relative abundance of species at class level (top 10); **(B)** The relative abundance of Coriobacteriia (class); **(C)** The relative abundance of species at order level (top 10); **(D)** The relative abundance of Coriobacteriales (order); **(E)** Relative abundance of species at family level (top 10); **(F)** The relative abundance of Coriobacteriaceae (family); **(G)** The relative abundance of species at the genus level (top 10); **(H–K)** Relative abundance of *Collinsella, Allobaculum, Oscillospira, Blautia* (genus). Note: compared with group ND, ##: *p* < 0.01, ###: *p* < 0.001; Compared with group HFD, *: *p* < 0.05.

The relative abundance of intestinal microbial communities in each group at class level was shown in Figures 7A, B. Compared to the ND group, the HFD group significantly affected the dominant intestinal bacteria such as Fusobacteriales, Bacilli, Erysipelotrichi, Bacteroidia, and Coriobacteriia. However, walnut oil intervention significantly reversed the non-dominant Coriobacteriia (*p* < 0.05).

The relative abundance of intestinal microbial communities in each group at the order level was shown in Figures 7C, D. Compared to the ND group, the HFD group significantly affected the dominant intestinal bacteria such as Fusobacteriales, Clostridiales, Lactobacillales, Bacteroidales, and Coriobacteriales. However, walnut oil intervention led to a significant hinder effect of Coriobacteriales only (*p* < 0.05).

The relative abundance of intestinal microbial communities in each group at the family level was shown in Figures 7E, F. Compared to the ND group, the HFD group significantly affected the dominant intestinal bacteria such as Fusobacteriaceae, Lactobacillaceae, Lachnospiraceae, Erysipelotrichaceae and Coriobacteriaceae. However, walnut oil intervention only blocked Coriobacteriaceae increase significantly (*p* < 0.05).

The relative abundance of intestinal microbial communities at the genus level in each group was shown in Figures 7G, K. Compared to the ND group, there was a significant increase in the relative abundance of *Collinsella* and *Blautia* (*p* < 0.01) and a significant decrease in *Oscillospira* and *Allobaculum* (*p* < 0.01) in the HFD group. However, compared with HFD group, only *Collinslla* in OL group was significantly impeded (*p* < 0.05).

In conclusion, the HFD group exhibited a significantly higher relative abundance of Coriobacteria group (class, order, family) and *Collinsella* in the family than the ND group (*p < 0.01*), while the OL group showed a significantly lower abundance of above flora (*p* < 0.05), indicative of negative bacteria group. The positive effect on the bacteria community was only observed *Allobaculum* and *Oscillospira* at the generic level with less abundant in HFD group than ND group (*p* < 0.01) and OL group.

#### 3.5.5 Linear discriminant analysis effect size (LEfSe) analysis of intestinal flora of rats in each group

**FIGURE 8:**
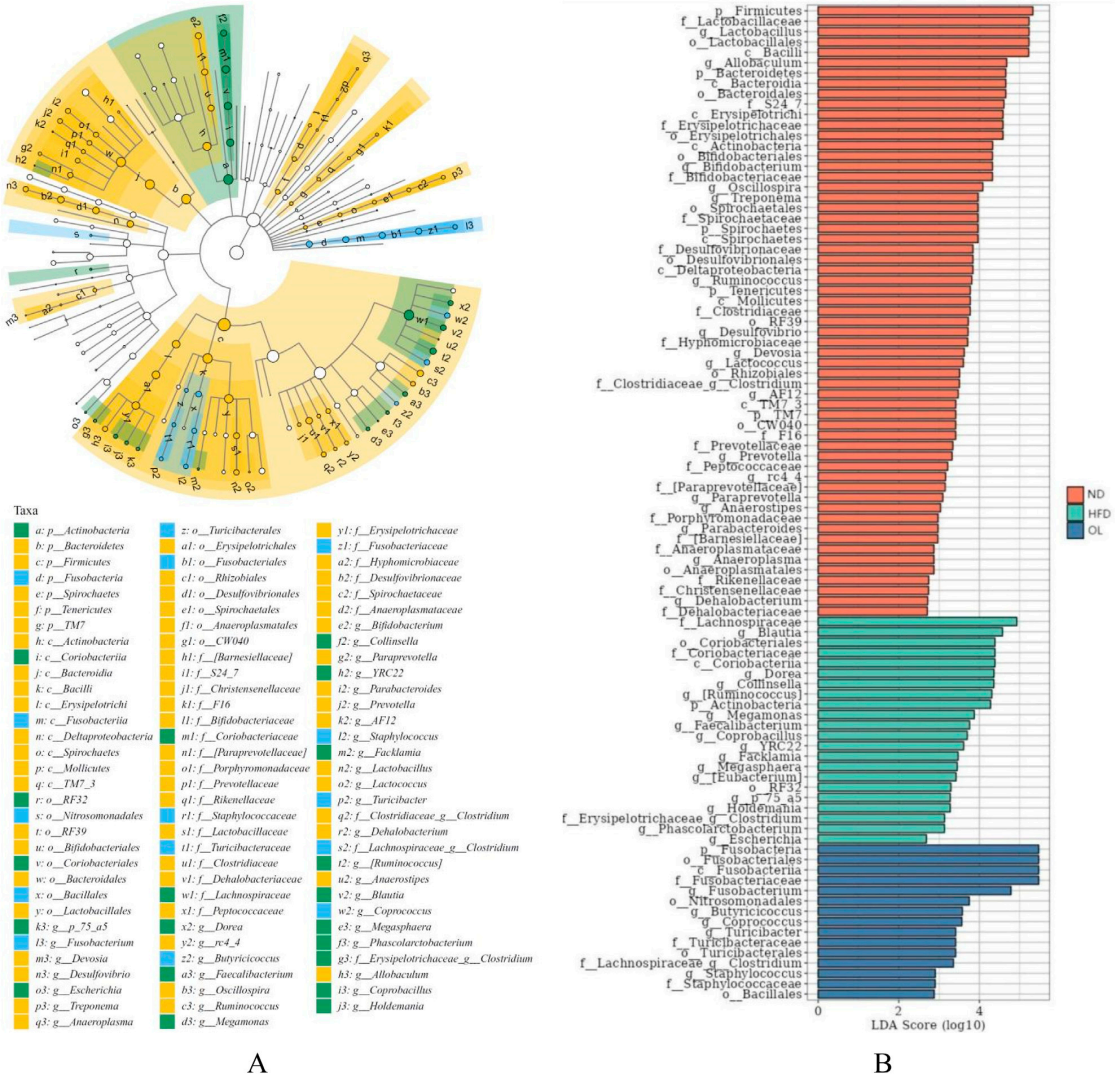
LEfSe results in the ND, HFD, and OL groups (n = 6). **(A)** LEfSe evolution of intestinal flora in each group; **(B)** Log LDA score of dominant biomarker taxa (threshold 2.5).

LEfSe analysis is a method combining non-parametric Kruskal-Wallis and Wilcoxon rank-sum tests with linear discriminant analysis (LDA) to identify features with biological significance (Nicola S et al., 2011). As shown in Figures 8A, B, with LDA > 2.5 as the screening threshold, the characteristic bacteria of rats in ND group were identified as Firmicutes and Lactobacillaceae, Lactobacillales and Bacilli. In contrast, the HFD group was characterized by Lachnospiraceae, *Blautia,* Proteobacteria and Coriobacteriaceae, while the OL group was characterized by Fusobacteria.

### 3.6 Liver metabolomics analysis

#### 3.6.1 Orthogonal partial least squares discriminant analysis (OPLS-DA) of the metabolome data

**FIGURE 9:**
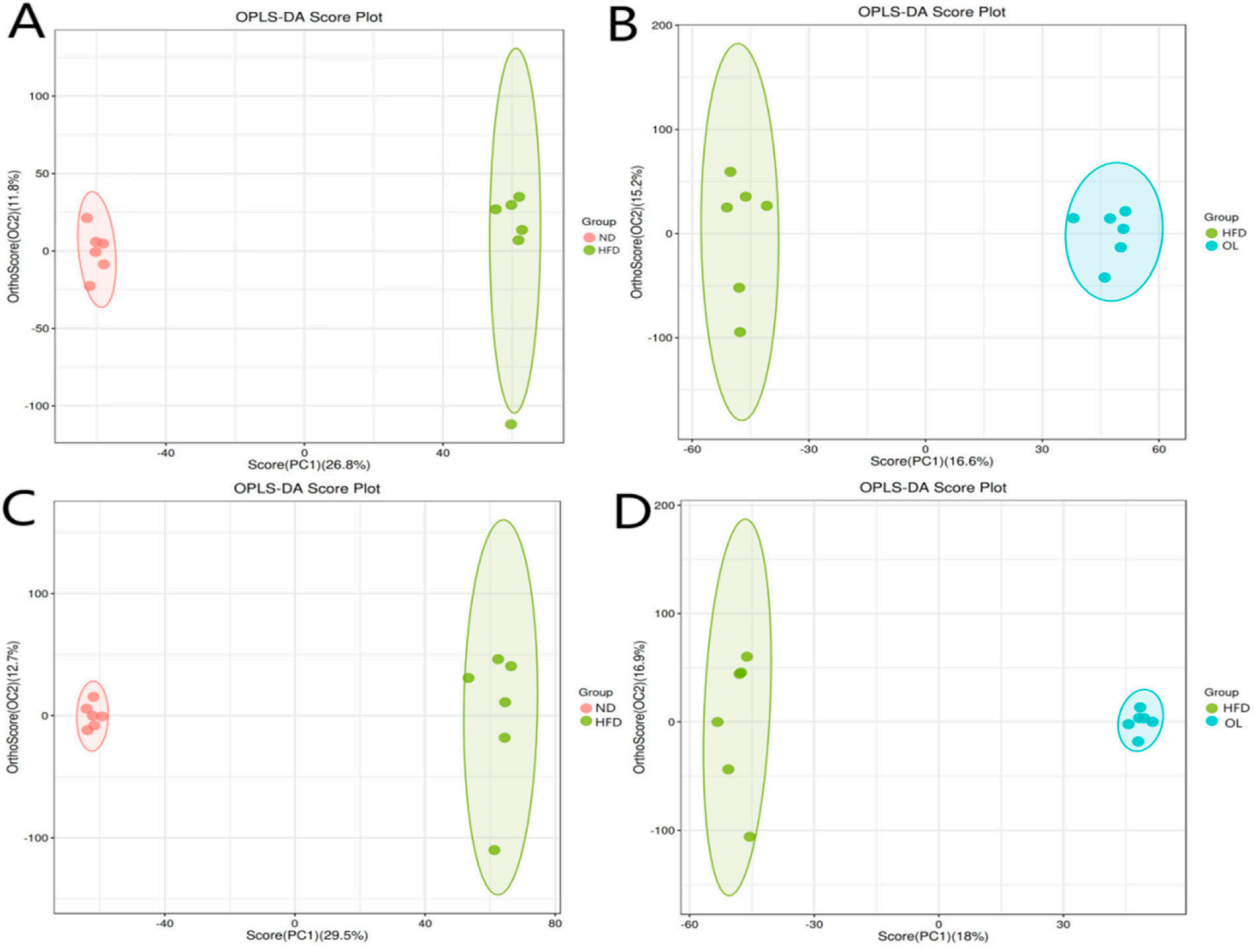
Ion patterns of OPLS-DA in liver samples. **(A, B)** Positive ion mode; **(C, D)** Negative ion mode.

Given the multidimensionality and the collinearity of the metabolome data, traditional univariate analysis were insufficient for the comprehensive and precise data mining. Therefore, a multivariate statistical method, OPLS-DA modeling, was used to evaluate the differences among groups (Etienne et al., 2015). The results, as shown in Figure 9, demonstrated clear segregation in both positive and negative ion modes, indicating significant differences among groups. The validation parameters of OPLS-DA model were showed in Table 1. R^2^Y represented the goodness of fit ranging from 0 to 1, where 1 indicated perfect fit. Q^2^ represented the prediction ability of the model. Referring to R^2^Y = 0.999, Q^2^ = 0.874 in positive ion mode and R^2^Y = 0.997, Q^2^ = 0.904 in negative ion mode in the comparison between ND and HFD groups, the model was stable and effective, and could be used for the follow-up screening of differential metabolites (He et al., 2020).

**TABLE 1:** Detailed validation parameters of OPLS-DA model for liver samples.

Group	Positive ion mode		Negative ion mode	
	R^2^Y (cum)	Q^2^ (cum)	R^2^Y (cum)	Q^2^ (cum)
HFD vs. ND	0.999	0.874	0.997	0.904
HFD vs. OL	0.993	0.498	0.998	0.629

#### 3.6.2 Identification of differential metabolites

**FIGURE 10:**
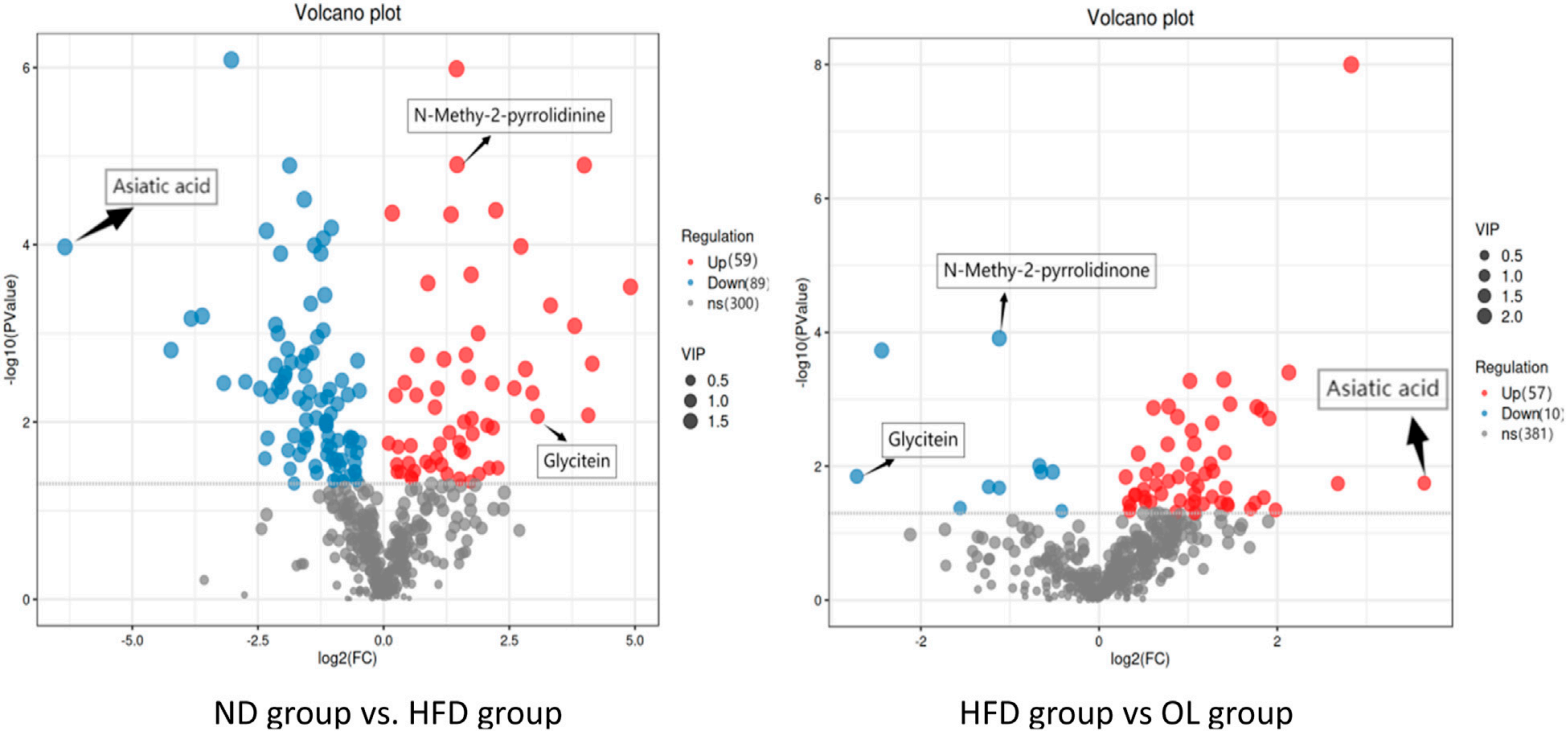
Volcanic maps of metabolites in model group and administration group.

Based on the difference analysis of all metabolites detected by OPLS-DA, a volcanic map was constructed (Figure 10) to observe the distribution of differential metabolism. Both Volcanic intensity parameter VIP > 1 and *p* < 0.05 were used as thresholds to screen differential metabolites (Yan et al., 2023). A total of 148 different metabolites were screened in HFD and ND groups, among which 59 metabolites were up-regulated and 89 metabolites were downregulated. A total of 67 different metabolites were screened in OL and HFD group, of which 57 metabolites were upregulated and 10 metabolites were downregulated.

Log_2_FC is the logarithmic transformation of the ratio of expression changes. A total of 19 potential biomarkers were screened out for the above differential metabolites with |log_2_FC| > 1 (Table 2), indicating the metabolite was more than 2 × up-regulated or less than 0.5 × down-regulated.

**TABLE 2:** Metabolite information of significant difference among groups.

name	Formula	KEGG	ND vs. HFD			HFD vs. OL		
			log_2_FC	-logP	VIP	log_2_FC	-logP	VIP
Glycitein	C_16_H_12_O_5_	C14536	3.06↑	2.07^##^	1.40	−2.72↓	1.85^*^	1.64
N-Methyl-2-pyrrolidinone	C_5_H_9_NO	C11118	1.34↑	4.34^####^	1.76	−1.12↓	3.91^***^	2.13
Spermidine	C_7_H_19_N_3_	C00315	−1.12↓	1.73^#^	1.22	1.47↑	2.93^**^	1.89
D-Xylonate	C_5_H_10_O_6_	C00502	−1.20↓	4.07^####^	1.67	1.02↑	3.27^***^	1.98
L-2-Amino-6-oxoheptanedioate	C_7_H_11_NO_5_	C03871	−1.25↓	3.91^###^	1.71	1.07↑	2.33^**^	1.92
N-Acetylmuramate	C_11_H_19_NO_8_	C02713	−1.42↓	2.78^##^	1.48	1.02↑	1.42^*^	1.38
Methylselenopyruvate	C_4_H_6_O_3_Se	C18904	−1.47↓	2.34^##^	1.48	1.05↑	1.81^*^	1.70
L-Arogenate	C_10_H_13_NO_5_	C00826	−1.54↓	2.75^##^	1.55	1.41↑	2.20^**^	1.76
Mannitol	C_6_H_14_O_6_	C00392	−1.58↓	4.51^####^	1.77	1.28↑	1.93^*^	1.79
Carnosine	C_9_H_14_N_4_O_3_	C00386	−1.63↓	2.67^##^	1.47	1.77↑	2.88^**^	1.94
17a-Estradiol	C_18_H_24_O_2_	C02537	−1.78↓	1.85^#^	1.45	1.70↑	1.36^*^	1.43
Sedoheptulose	C_7_H_14_O_7_	C02076	−1.87↓	4.89^####^	1.68	1.91↑	2.71^**^	1.89
Pantothenic acid	C_9_H_17_NO_5_	C00864	−1.95↓	2.55^##^	1.45	1.82↑	2.84^**^	1.89
L-Lysine	C_6_H_14_N_2_O_2_	C00047	−1.97↓	2.51^##^	1.64	1.17↑	1.44^*^	1.49
Nonadecanoic acid	C_19_H_38_O_2_	C16535	−2.05↓	3.90^###^	1.64	1.04↑	2.53^**^	1.78
Hippuric acid	C_9_H_9_NO_3_	C01586	−2.33↓	4.16^####^	1.76	1.27↑	1.55^*^	1.47
6-Thiourate	C_5_H_4_N_4_O_2_S	C16613	−2.36↓	1.59^#^	1.20	2.13↑	3.40^***^	2.02
Norlinolenic acid	C_17_H_28_O_2_	C16344	−3.61↓	3.20^###^	1.77	1.44↑	1.44^*^	1.54
Asiatic acid	C_30_H_48_O_5_	C08617	−6.34↓	3.97^###^	1.76	3.65↑	1.75^*^	1.59

Note: compared with group ND, #: *p < 0.05,*##: *p* < 0.01, ###: *p* < 0.001, #####: *p < 0.0001;* Compared with group HFD, *: *p* < 0.05, **: *p* < 0.01,***: *p* < 0.001.

Significantly increased by 8.32-fold (log_2_FC = 3.06) and 2.53-fold (log_2_FC = 1.34) in the HFD group (*p* < 0.01 and *p* < 0.0001), but were remarkedly blocked to 0.15 times (log_2_FC = −2.72) and 0.46 times (log_2_FC = −1.12) after walnut oil treatment (*p < 0.05* and *p < 0.001*). Other 17 biomarkers, including L-lysine, methylselenopyruvate, mannitol, L-2-amino-6-oxoheptanedioate, demethanate norlinolenic acid, 17a-estradiol, spermidine, D-xylonate, hippuric acid, 6-thiourate, L-arogenate, sedoheptulose, pantothenic acid, carnosine, N-acetylmuramate, nonadecanoic acid, and asiatic acid, were significantly decreased in the HFD group to less than 46% (*p < 0.05*), yet were significantly increased by 2.02 times after walnut oil treatment (*p < 0.05*), as detailed in Figure 10 and Table 2. These biomarkers were predominantly involved in lipid metabolism, indicating that walnut oil had a strong alleviative or mitigatory effect on metabolic disorders induced by high-fat diet.

#### 3.6.3 Kyoto encyclopedia of genes and genomes (KEGG) metabolic pathway analysis

**FIGURE 11:**
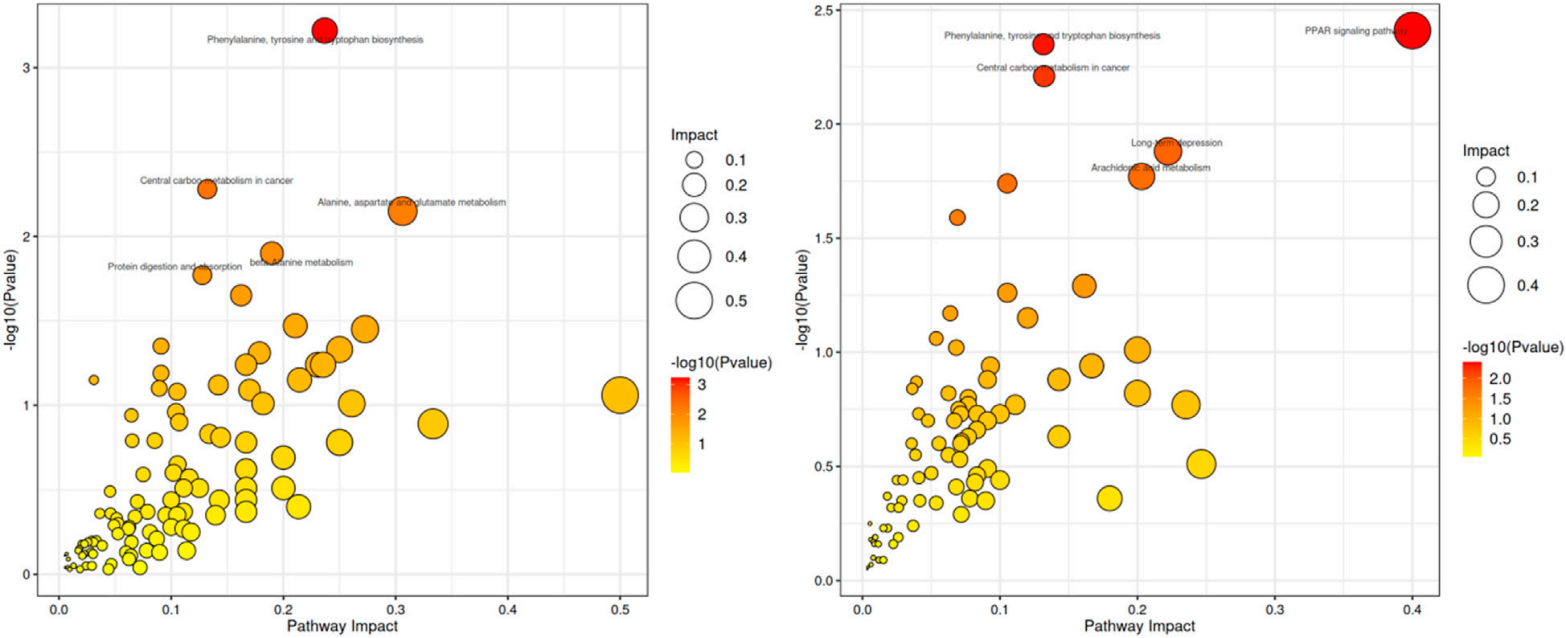
Scatterplot of metabolic pathway influencing factors in HFD vs. ND (left) and OL vs. HFD (right).

KEGG pathway enrichment analysis helps to identify the primary metabolic pathways and signaling pathways involved in the differential metabolites (Li et al., 2023). MetaboAnalyst (www.metaboanalyst.ca) was used to perform KEGG pathway enrichment analysis on the list of differential metabolites (Jianguo and David, 2011). The analysis revealed that, compared to the ND group, the HFD group showed significant enrichment in pathways such as phenylalanine, tyrosine and tryptophan biosynthesis, central carbon metabolism in cancer, alanine, aspartate and glutamate metabolism. Compared to the HFD group, the OL group was significantly enriched in Peroxisome Proliferator-Activated Receptors (PPAR) signaling, phenylalanine, tyrosine and tryptophan biosynthesis, central carbon metabolism in cancer and other metabolic pathways (Figure 11).

### 3.7 Correlation analysis of intestinal flora, metabolomics and biochemical indexes

**FIGURE 12:**
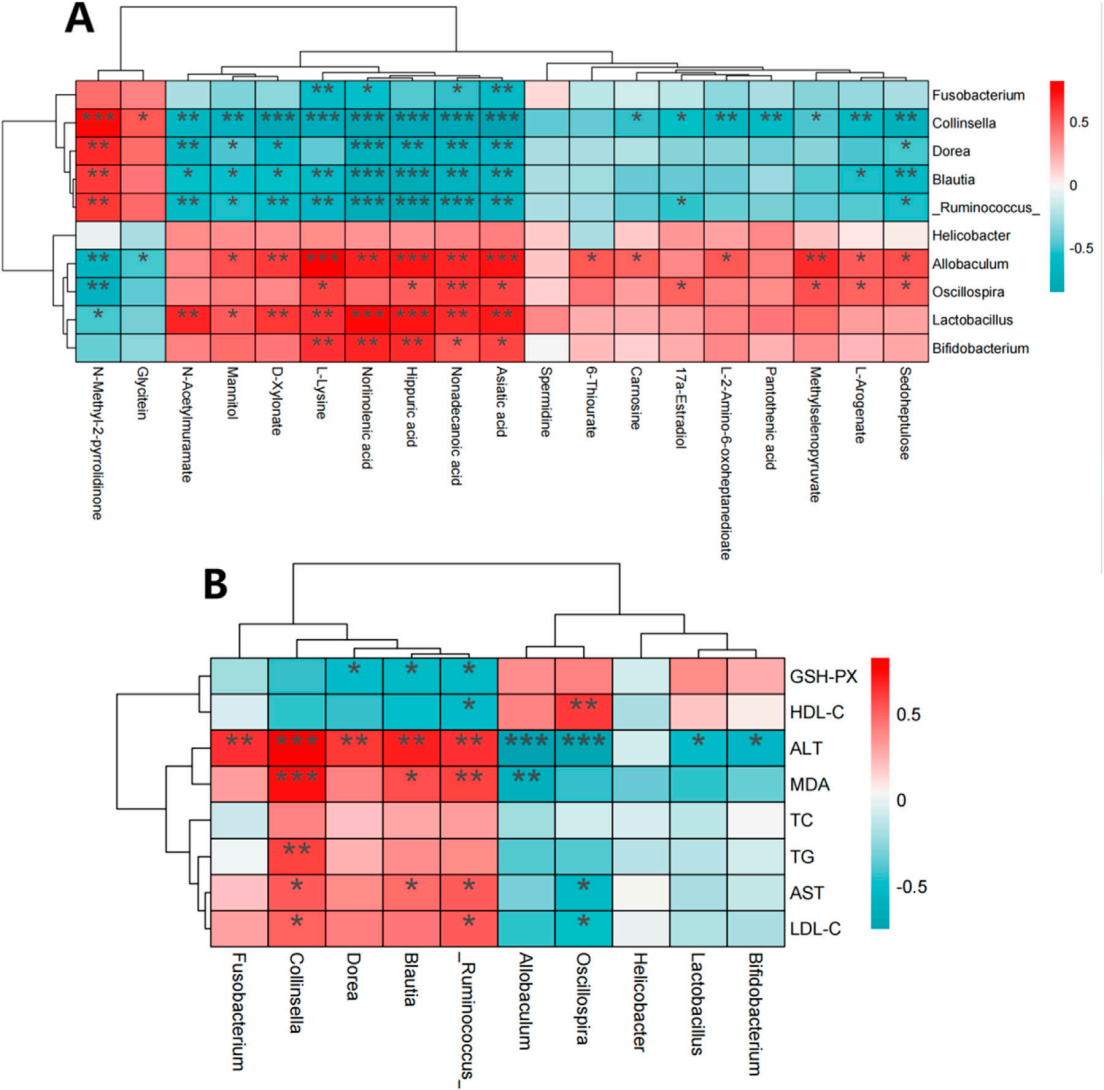
Spearman correlation between changes in gut microbiota and metabolomics or physiological and biochemical indicators. **(A)** Spearman correlation between differential metabolites and gut microbiota; **(B)** Spearman correlation between gut microbiota and physical and chemical indexes. Note: Red indicates positive correlation and green indicates negative correlation. *: *p* < 0.05, **: *p* < 0.01, ***: *p* < 0.001, ****: *p* < 0.0001.

To identify the interactions between liver metabolites and intestinal flora, a Spearman correlation analysis was conducted on differential metabolites and key bacterial genera, including *Lactobacillus, Fusobacterium, Allobaculum, Blautia, Collinsella, Oscillospira, Helicobacter,* (Figure 12A). Some liver metabolites were found significantly correlated with the intestinal flora. *Collinsella, Ruminococcus, Blautia* and asiatic acid, nonadecanoic acid, hippuric acid, norlinolenic acid, L-lysine, D-xylonate, mannitol, N-acetylmuramate were negatively correlated, while *Allobaculum*, *Lactobacillus* and asiatic acid, nonadecanoic acid, hippuric acid, norlinolenic acid, L-lysine were positively correlated. *Ruminococcus, Blautia, Dorea, Collinsella* and *Fusobacterium* were positively correlated with ALT, MDA, TC, TG, AST and LDL-C in rats. GSH-PX and HDL-C were negatively correlated. However, *Bifidobacterium, Lactobacillus, Helicobacter, Oscillospira* and *Allobacuium* were inversely correlated with the above indicators of rats, as shown in Figure 12B.

### 3.8 Correlation analysis of intestinal flora, metabolomics, and physiological and biochemical indice

**FIGURE 13:**
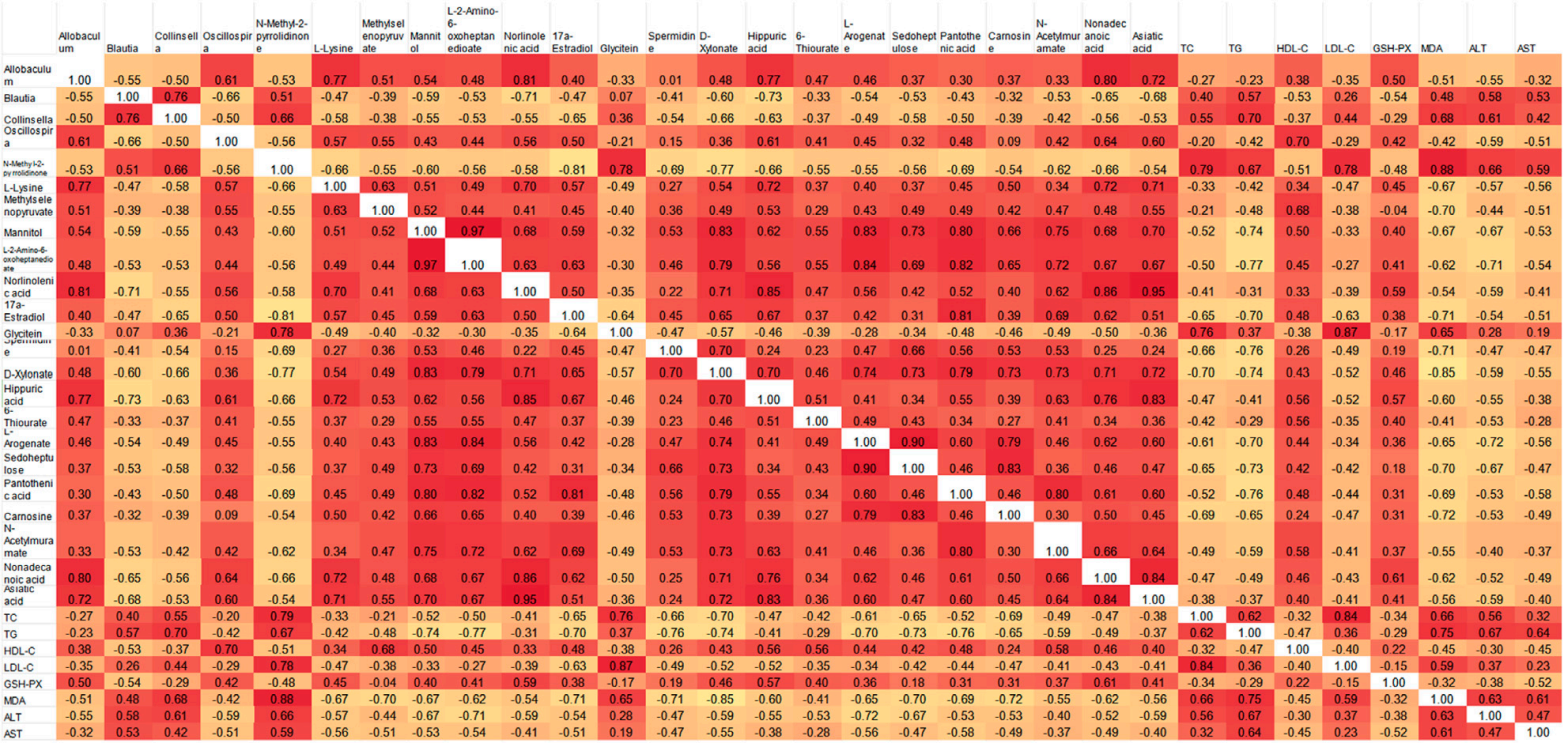
Pearson correlation heat map of intestinal microbiota, metabolomics and physicochemical indexes.

Spearman correlation analysis was used to further clarify the interaction among liver metabolites, gut microbiota, and physiological and biochemical indicators. The analysis included key bacteria such as *Allobaculum, Blautia, Collinsella,* and *Oscillospira* along with differential metabolites indicators (Figure 13). The results indicated that gut microbiota, liver metabolites and physiological and biochemical indicators were significantly correlated. *Allobaculum* and *Oscillospira* showed positive correlations with metabolites like L-lysine, methylselenopyruvate, mannitol, L-2-amino-6-oxoheptanedioate, norlinolenic acid, 17a-Estradiol and other metabolites, and negative correlations with TC, TG, LDL-C, MDA, ALT, and AST. Conversely, for *Blautia and Collinsella* the correlation was completely in an opposite way. These results suggested that gut flora might affect liver metabolites and physiological and biochemical markers of hyperlipidemia.

## 4 Discussion and conclusion

In this study, serum assay showed that the levels of TC, TG and LDL-C significantly decreased in all walnut oil treatment groups (high, medium and low doses), suggesting a potent lipid-lowering effect of walnut oil. *Coriobacteriaceae* and *Collinsella* in the HFD group were significantly increased (*p* < 0.01) against ND group, while OL group showed a notable reduction compared to HFD group (*p* < 0.05). *Collinsella* has been reported to be positively correlated with serum LDL-C and liver TG, TC, and negatively correlated with serum HDL-C and intestinal short-chain fatty acids, such as acetic acid, propionic acid, and butyric acid (Lu et al., 2022). These findings are consistent with the results of this study. The positive effector flora was only reflected in *Allobaculum* at the genus level, which was basically consistent with existing literature (Li et al., 2024). The relative abundance of *Oscillospira* was significantly decreased in the HFD group (*p* < 0.01), however the OL group displayed a relative increase, indicating its role as a beneficial bacteria. These observations were in agreement with a previous study (Yang et al., 2021). The relative abundance of *Allobaculum* was significantly increased in our study (*p < 0.01*), suggesting a negative correlation with inflammation, insulin resistance and obesity, which were congruent with the prior investigation (Zheng et al., 2021). The increase of *Allobaculum* also has an inhibitory effect on lipid absorption, affirming its role as a positive influence (Conor et al., 2019). After the walnut oil intervention, the trends of changes for some metabolites were blocked. Pathway analysis indicated that these metabolites were mainly related to PPAR signaling pathway and the biosynthesis of phenylalanine, tyrosine and tryptophan. Furthermore, Spearman and Pearson correlation analysis demonstrated that asiatic acid, nonadecanoic acid and hippuric acid, norlinolenic acid, L-lysine were significantly positively correlated with *Allobaculum, Lactobacillus, etc.*

In conclusion, walnut oil significantly mitigated the rise of blood lipids in hyperlipidemic rats induced by a high-fat diet. It could regulate the disorder of intestinal flora, increase the relative abundance of beneficial intestinal flora, and improve liver metabolism in hyperlipidemic rats. This study provided a reference for further research on the preventive and interventive potentiality of walnut oil in hyperlipidemia and the high-value utilization of walnut kernel.

## Data Availability

The datasets presented in this study can be found in online repositories. The name of the repository and accession number can be found below: NCBI, SAMN46168266.
